# Cultural adaptation of two school-based smoking prevention programs in Bogotá, Colombia

**DOI:** 10.1093/tbm/ibab019

**Published:** 2021-04-26

**Authors:** Sharon Sánchez-Franco, Luis Fernando Arias, Joaquin Jaramillo, Jennifer M Murray, Ruth F Hunter, Blanca Llorente, Linda Bauld, Sally Good, Judith West, Frank Kee, Olga L Sarmiento

**Affiliations:** 1 Department of Public Health, School of Medicine, Universidad de Los Andes, Bogotá, Colombia; 2 Centre for Public Health, Institute of Health Sciences, School of Medicine, Dentistry and Biomedical Sciences, Queen’s University Belfast, Belfast, UK; 3 Anaás Fund, Bogotá, Colombia; 4 The Usher Institute and SPECTRUM Consortium, College of Medicine and Veterinary Medicine, University of Edinburgh, Edinburgh, UK; 5 Evidence to Impact, Bristol, UK; 6 Cancer Focus, Belfast, UK

**Keywords:** Cultural adaptation, Smoking prevention, Adolescents, Tobacco control, Public health, Intervention

## Abstract

Smoking prevention among adolescents is a public health challenge that is even more significant in low- and middle-income countries where local evidence is limited and smoking rates remain high. Evidence-based interventions could be transferred to low- and middle-income country settings but only after appropriate cultural adaptation. This paper aims to describe the process of the cultural adaptation of two school-based smoking prevention interventions, A Stop Smoking in Schools Trial and Dead Cool, to be implemented in Bogotá, Colombia. A recognized heuristic framework guided the cultural adaptation through five stages. We conducted a concurrent nested mixed-methods study consisting of a qualitative descriptive case study and a quantitative pre- and post quasi-experiment without a control. Contextual, content, training, and implementation modifications were made to the programs to address cultural factors, to maintain the fidelity of implementation, and to increase the pupils’ engagement with the programs. Modifications incorporated the suggestions of stakeholders, the original developers, and local community members, whilst considering the feasibility of delivering the programs. Involving stakeholders, original program developers, and community members in the cultural adaptation of evidence-based interventions is essential to properly adapt them to the local context, and to maintain the fidelity of program implementation.

**Implications**

**Practice:** A systematic process for cultural adaptation of school-based smoking prevention programs, including a strategy for maintaining the fidelity, can be used in other countries of Latin America and the global south.

**Policy:** Evidence-based and culturally adapted strategies promote awareness of tobacco control within school environments, contributing to the global effort toward tobacco control.

**Research:** Future research should assess how effectively adapt smoking prevention programs in vastly different contexts to contribute to understanding the transferability and applicability of evidence.

## INTRODUCTION

Tobacco is a major risk factor for several noncommunicable diseases and causes 8.1 million global deaths annually [1]. Adolescence is the most critical life stage for tobacco consumption because: (i) young people are more susceptible to social influence [2]; (ii) 90% of smokers start smoking before they are 19 years old [3]; and (iii) adolescent smoking is associated with risky and violent behaviors [4]. Therefore, preventing tobacco consumption among adolescents is a priority for public health.

There are several strategies at the individual, community, and policy level to prevent and reduce smoking among youth, endorsed by the Framework Convention on Tobacco Control (WHO-FCTC) [4]. At the individual level, effective interventions include prevention programs in pediatric clinical practice and cessation programs [5, 6]. At the community level, strategies have focused on mass media campaigns, changing social norms, and institutional policies [4, 6]. At the school level, smoking prevention is based on social influence, cognitive-behavioral skills, life skills, and enhancing social competence [4, 7]. At the policy level, effective strategies include taxation of tobacco, bans on advertising, promotion and sponsorship, plain packaging and labeling, and regulation of smoke-free areas [4, 6]. There is also a consensus in the literature that all community and policy strategies could directly impact on prevention at the individual level [4, 6, 7].

Smoking prevention is a public health challenge that is even greater in low- and middle-income countries (LMICs) where the local evidence is limited [4] and rates of smoking remain high [8, 9]. Additionally, the tobacco industry has focused on LMICs as its markets are eroded elsewhere [10]. Colombia reflects many of the typical practices related to adolescent smoking in Latin America (LA) [9]. In 2015, Colombia had the fifth highest prevalence of daily tobacco use among children in LA (10–14 years old) [11]. Thus, it represents a very relevant context for adapting and investigating interventions that have been proven to be effective elsewhere at reducing adolescent smoking uptake rates [12, 13]. However, these interventions need to be adapted for upscaling to other LMICs.

In this context, the *MECHANISMS* study aims to improve the measurement of social norms for smoking behaviors in adolescents in Northern Ireland (UK) and Bogotá (Colombia), and to characterize the mechanisms of action of two smoking prevention interventions in schools: A Stop Smoking in Schools Trial (ASSIST) and Dead Cool [14]. The *MECHANISMS* study required the cultural adaptation of each of the interventions, accounting for differences and common factors existing between both settings while maintaining the fidelity of the implementation of the programs. Both Northern Ireland and Colombia have a historical context of civil conflict. However, Northern Ireland is classified as a high-income country with approximately 2 million inhabitants, whilst Bogotá is the capital city of Colombia and has over 7.2 million inhabitants [15–17]. This study aims to describe the process of the cultural adaptation of the two school-based smoking prevention interventions to be implemented in Bogotá.

### The interventions

ASSIST and Dead Cool offered several distinct advantages for studying mechanisms by which the programs could change social norms but we recognized that these mechanisms could be contingent on local context in Bogotá and their triggering would require initial cultural adaptation. First, both interventions have been shown to be effective for preventing smoking onset among school children in cluster randomized trials conducted in the UK [12, 13]. Second, the programs are based on different behavioral change strategies. ASSIST focuses on leveraging social networks to spread antismoking social norms and represents an innovative attempt at harnessing positive peer influence. Dead Cool is based on the Theory of Planned Behaviour and uses conventional classroom pedagogy to promote social and personal skills learning. In the *MECHANISMS* study, the different approaches used by these programs represent relevant contrasting contexts for assessing the mechanisms underlying the influence of social norms on adolescent smoking behaviors in different settings [14]. Furthermore, both interventions are relevant for the local context as they fit well into the health education curriculum of schools in Bogotá and invoke different intrapersonal and interpersonal influences on smoking behavior, to varying extents [18].

In ASSIST, all pupils nominate up to five classmates whom they view as influential. The top 18% of nominated pupils are trained to be peer supporters in a 2-day course at a venue away from the school [12]. The training aims to increase peer supporters’ knowledge about the health, economic, social, and environmental risks of smoking, to emphasize the benefits of remaining smoke-free, and to develop skills to promote nonsmoking among their peers. After the training, the peer supporters intervene informally in everyday situations to encourage their peers not to smoke and use a diary to record their conversations. Four follow-up visits are made by the ASSIST trainers over 10 weeks to provide further training and to monitor their progress [12]. Schools participating in ASSIST are often chosen based on high deprivation and high smoking prevalence, and the cost of the program is covered by local authorities rather than the schools themselves.

Since the original Randomized Controlled Trial, Dead Cool had been undated to emphasize skills development based on the Behaviour Change Taxonomy [13, 19]. The program examines the influences on smoking behavior from friends, family members, and communication and media. It consists of eight lesson plans delivered by teachers from the school in their classes over 8 weeks. Teachers are provided with a resource pack including a DVD of short videos, lesson guides, fact files, and PowerPoint slides for each lesson. Pupils are provided with pupil workbooks. Before the program, the pupils have an introductory session and teachers receive professional instruction where the focus and epistemology behind the program are outlined [13]. Dead Cool is offered free of charge to all postprimary schools where it meets the official curriculum’s needs.

### Study setting

Both evidence-based interventions were developed and tested in the UK, where the social norms, culture, and smoking behavior differ from those in Colombia. Colombia is a Spanish-speaking country with important ethnic minorities including afro and indigenous populations (14.8%) [17]. Ethnic minorities in the UK are also significant as a result of modern-era immigration (13%) [16]. Social differences between the countries are apparent in terms of absolute poverty with a rate of 4.1% in Colombia and 0.2% in the UK [15]. The educational systems and outcomes are also very different; for example, there are 26 pupils per teaching staff in secondary public institutions in Colombia, and only 17 in the UK [15]. In addition, 36% of Colombian students and 6% of UK students are rated at the lowest mathematics proficiency on the Program for International Student Assessment (PISA) [15].

Whilst both countries have partially fulfilled their commitments under WHO-FCTC, they have different smoking prevalence rates. In particular, Colombia has faced obstacles in implementing WHO-FCTC (e.g., adolescents still have access to tobacco products, and consumption persists). In Colombia, the smoking rates among adolescents aged 13–15 years are up to 6 percentage points higher than in the UK (12% in boys and 9% in girls in Colombia; 6% in boys and 8% in girls in the UK) [11]. In 2016, 28.3% of Colombian students aged 12–18 years had consumed cigarettes at least once in their life, and they started to smoke at 12.9 years old on average [20]. Colombia has an intersectoral policy to promote healthy school environments focused on the pupils’ physical and mental health [21]. However, the implementation of these policies has generated challenges in fitting the health aims to the education system’s characteristics. For example, schools with public funds have access to fewer resources and constantly underperform in standardized tests [22].

### Framework for cultural adaptation

Cultural adaptation of evidence-based interventions is necessary when attempting to translate them to a new population [23]. It implies modifications intended to improve an intervention’s fit in a specific context and/or subcultural groups while preserving the fidelity of the core elements [24], but it can also improve the reach, engagement, effectiveness, and sustainability of programs while reducing health inequalities [25].

The extant literature offers various frameworks for culturally adapting evidence-based interventions [26]. The Framework to Report Adaptation and Modifications-Expanded (FRAME) provides a guide for conceptualizing the different sorts of modifications that may be part of an adaptation and links together their process, nature, and outcomes [24, 26]. This framework reflects the complex and dynamic settings encountered in the implementation of interventions in vastly different contexts, which may facilitate or impede the scale-up and sustainability of the interventions [24]. It is particularly useful to replicate the cultural adaptation process in different populations because this facilitates the systematic reporting and analysis of the modifications [24]. FRAME is the update of Wiltsey Stirman et al.’s proposed framework and coding system [27] that draws upon a systematic review of frameworks and cultural adaptations of interventions in the USA and Latin, European, and Asian countries [24]. FRAME employs eight different aspects that detail what adjustments were made to the original version, where they were made, when they were made, who they were made by, and why they were made [24, 27].

## METHODS

### Cultural adaptation process

We conducted a concurrent nested mixed-methods study consisting of a qualitative descriptive case study and a quantitative pre- and post quasi-experiment without a control group. We culturally adapted the interventions simultaneously, from April 2018 to December 2019, using the five stages of the heuristic framework proposed by Barrera and Castro [28, 29]. Supplementary Appendix 1 shows examples of the activities and participants.

#### Step 1: information gathering

A local stakeholder committee was formed to outline the aims of an adaptation plan, according to local contextual factors. Additionally, representatives of Bogotá’s research team visited Northern Ireland to receive training on both programs from their developers, after reviewing the manuals and activities.

#### Step 2: preliminary adaptation design

Adaptation materials were drafted according to the adaptation plan, and included three components. First, bilingual research assistants translated all materials from English to Spanish. The videos that are part of the Dead Cool program were dubbed using voice actors with Colombian accents. Second, a “mock-up” version was proposed including the suggestions of the stakeholder committee. Third, a bilingual researcher conducted a back-translation of all adapted material using interpretative sense-checking. When a discrepancy between the English and Spanish versions was detected, researchers, intervention developers, and the local committee discussed modifications until an agreement was reached.

#### Step 3: preliminary adaptation test

We tested the adaptations in two schools selected with the following inclusion criteria: (i) schools in the urban area; (ii) including boys and girls; (iii) having enrolled between 90–150 pupils in seventh year. From 13 schools, two accepted the invitation and were randomly assigned to each of the interventions. In total, 239 pupils participated in the pilot (142 in ASSIST and 97 in Dead Cool).

The qualitative data included four instruments to identify the difficulties and possible solutions in the implementation protocol. First, the individual practitioners completed a field diary during each activity (*N* = 37 diary files). Second, we conducted an interview and a focus group with all individual practitioners (*N* = 3). Third, we conducted four focus groups with pupils (*N* = 25). Fourth, to assess the fidelity of the implementation of the programs, the original program developers visited Bogotá to assess their delivery, using direct observation of previously selected activities, and then provided feedback. Up to three intervention sessions were also videotaped to check intervention fidelity. The quantitative data included a self-administered survey to collect sociodemographic information, smoking behavior and intentions, knowledge of smoking, other smoking behavior mediators, and a social network questionnaire. In addition, each student completed a behavioral economics (Game Theory) experiment to identify social norms related to smoking. Further details on all of the study’s measurement instruments are available in Hunter et al. [14]. Qualtrics software was used to collect data (Qualtrics, Provo, UT, Version Jan. 2019).

#### Step 4: adaptation refinement

We refined the intervention procedures and materials according to the results of steps 1–3.

#### Step 5: cultural adaptation trial

We conducted a trial of the outcomes of the prior steps (within the full phase of the *MECHANISMS* study) to assess the fidelity of the implementation of the programs, the expected outcomes, and the engagement of the pupils. We applied the study design (from step 3) in 6 schools sampled using the following process: first, 40 schools were prioritized by the local Education and Health offices based on health risks; second, we invited 13 schools to participate based on the same inclusion criteria used in step 3; third, of the 6 schools that accepted the invitation, 3 were randomly assigned to ASSIST (340 pupils), and 3 were assigned to Dead Cool (314 pupils). In total, 654 pupils participated in this step.

In this step, we collected qualitative data using 2 focus groups with all individual practitioners (*N* = 4) and 10 focus groups with pupils (*N* = 56). The quantitative data include pupils’ attendance records, and records of the implementation of activities in each session. Before and after the interventions, we also collected the same self-administered survey and experiments from step 3. In this paper, we have reported data on the knowledge of smoking, smoking behavior, and the acceptability of the programs. Knowledge was assessed with six questions [14, 30], and the total score of correct answers was dichotomized into high knowledge (score ≥3) and low knowledge (score <3). Current and past smoking behavior was measured using four questions, which were recoded into three categories: current smoker, previous smoker, and never smoker [14]. Acceptability was measured with the question “Do you think your school has given you enough information on smoking?” [14, 31].

### Data analysis

The qualitative data were analyzed using thematic analysis through two cycles of coding. The first coding cycle identified suggestions for modifications regarding the materials and practical issues. The second coding cycle identified patterns through a top-down coding method, guided by the FRAME approach [24, 27]. We also identified patterns of participants’ engagement in both programs. The coding team employed multiple intercoder review techniques to establish intercoder reliability, including debriefing and member checking. NVivo qualitative data analysis software was used (QSR International Pty Ltd., Version 12, 2018).

According to the FRAME approach, we used four structural categories. First, contextual modifications refer to changes that define the way the overall interventions were delivered [27]. Second, content modifications refer to changes in the delivery of the interventions [27]. Third, we included changes in the components of training and evaluation [24]. Fourth, we included strategies to implement and disseminate the interventions [24]. Additionally, we identified the following five aspects for each adaptation: when the modification was made, whether the modification was planned or reactive, who participated in the modification, the adaptation goals, and the adaptation reasons [27].

For the quantitative data, we conducted a descriptive analysis of the baseline sociodemographic characteristics, smoking behavior, and intentions to smoke. Within the cultural adaptation trial (step 5), we calculated the attendance and implementation rate and the proportion of pupils who started to smoke after the interventions. Furthermore, we conducted a repeated-measures logistic regression analysis (“xtgee” command), with individual participants clustered within school classes, to identify changes in knowledge of smoking after the interventions, as an expected short-term outcome. Odds ratios (ORs) and 95% confidence intervals (CIs) are reported. These models were adjusted for the pupils’ sociodemographic characteristics (age, sex, socioeconomic level, and ethnicity). The statistical package Stata was used (StataCorp, 2015; Stata Statistical Software: Release 14; College Station, TX: StataCorp LP).

## RESULTS

### Findings of the information gathering (step 1)

The local stakeholder committee included: (i) an expert in public health and economics with 14 years of experience on assessing tobacco control policies in Colombia and LA; (ii) an expert in psychological therapy with 6 years of experience on psychoactive substance use prevention in adolescents in Colombia; and (iii) an expert in health behavior with 5 years of experience working with schools and children in Colombia. The committee focused on delineating and accommodating the cultural adaptation plan according to the characteristics of the target population who were students of public schools in Bogotá.

As a result of the first step, the committee defined the goals of the adaptation: (i) to address cultural factors like language, values, and policies on the implementation of the programs in Bogotá; (ii) to maintain the fidelity of the programs in a new target population; (iii) to increase engagement of the participants in the two new programs; and (iv) to assess potential culturally competent Spanish names for each intervention. Consequently, ASSIST was changed to the culturally adapted name: *Entre Parceros* (Among Pals) and Dead Cool was adapted to *Bacanísimo* (Very Cool).

### Findings of the adaptation steps (steps 2–4)

Modifications were made to both programs across the preliminary adaptation design (step 2), preliminary adaptation test (step 3), and adaptation refinement (step 4) steps. Table 1 presents the sociodemographic characteristics of the participants taking part in step 3. In general, a similar proportion of boys and girls participated in the study. Most of the pupils were aged between 11 and 14 years old, lived in a household with both parents and had mothers with only a secondary level education or less.

**Table 1:** Baseline characteristics of participants in the preliminary adaptation test (step 3) and adaptation trial (step 5)

	Step 3: preliminary adaptation test			Step 5: adaptation trial		
	ASSIST	Dead Cool		ASSIST	Dead Cool	
	*n* (%)	*n* (%)	*p* ^c^	*n* (%)	*n* (%)	*p* ^c^
Total	142	97		340	314	
Sex						
Boy	64 (45.07)	48 (52.17)	.288	175 (51.62)	156 (50.00)	.679
Girl/prefer not to say	78 (54.93)	44 (47.83)		164 (48.38)	156 (50.00)	
Age						
11–12 years old	71 (50.00)	55 (59.78)	.143	122 (35.88)	101 (32.17)	.316
13–14 years old	64 (45.07)	33 (35.87)	.163	195 (57.35)	170 (54.14)	.408
15 and over years old	7 (4.93)	4 (4.35)	.837	23 (6.77)	43 (13.69)	.003
Ethnic minority^a^	25 (17.61)	5 (5.43)	.006	41 (12.09)	50 (16.3)	.148
Home composition						
Single parent	43 (31.16)	40 (44.44)	.042	132 (39.64)	124 (39.87)	.952
Both parents	92 (66.67)	45 (50.00)	.012	178 (53.45)	167 (53.70)	.950
Other adults	3 (2.17)	5 (5.56)	.175	23 (6.91)	20 (6.43)	.809
Socioeconomic level^b^						
Low (<3)	139 (97.89)	89 (91.75)	.026	188 (55.29)	155 (49.36)	.129
Middle (3–5)	3 (2.11)	8 (8.25)		152 (44.71)	159 (50.64)	
Mother’s education level						
None/elementary	77 (54.23)	42 (48.84)	.430	120 (37.62)	76 (26.48)	.003
Secondary	49 (34.51)	30 (34.88)	.953	12 2(38.25)	114 (39.72)	.710
Postsecondary	11 (7.75)	12 (13.95)	.132	73 (22.88)	84 (29.27)	.073
Unknown	5 (3.52)	2 (2.33)	.612	4 (1.25)	13 (4.53)	.015
Smoking behavior						
Current smoker	7 (5.07)	9 (10.00)	.154	10 (3.00)	5 (1.61)	.241
Previous smoker	18 (13.04)	15 (16.67)	.447	60 (18.02)	54 (17.36)	.828
Never smoking	113 (81.88)	66 (73.33)	.124	263 (78.98)	252 (81.03)	.516
Intention to smoke	66 (47.83)	47 (52.22)	.516	137 (41.14)	122 (39.23)	.621
Knowledge of smoking						
High	18 (12.68)	16 (16.49)	.407	41 (12.06)	80 (25.48)	<.001
Low	124 (87.32)	81 (83.51)		299 (87.94)	234 (74.52)	

*Note*: Sample sizes may not sum to total *N* due to missing data.

^a^Includes indigenous, afro, and gipsy.

^b^An official six-level measurement that includes external characteristics of housing.

^c^*p* value of chi-square test for independence of dichotomized variables and interventions.

According to FRAME, we have reported the modification of both prevention programs using four categories (context, content, training and evaluation, and implementation and scale-up). Additionally, we have detailed the steps during which the changes occurred, their goals, and who participated in the decision making. Table 2 provides an overview of the key changes made to ASSIST and Dead Cool.

**Table 2:** Overview of the modifications to culturally adapt ASSIST (A) and Dead Cool (D)

Original version		Adapted version	What is the nature?	When did it occur?	Planned or reactive?	Who participated?	Adaptation goals	Adaptation reasons
Changes of context								
A	Peer supporter training delivered outside of school in venues like hotels, conference centers, or sports clubs	Peer supporter training was conducted in rooms of a university, using campus facilities	Setting	Preliminary adaptation design and adaptation refinement	Planned	Researchers, program manager, intervention developers	To reduce costs and to preserve intervention outcomes	Available space, resources and location, and accessibility of the venue
D	Delivered by teachers from the schools	Delivered by professionals hired directly	Personnel	Preliminary adaptation test	Reactive	Researchers, program manager, community members	To address cultural factors and to improve the feasibility	Teachers’ available time resources and compliance, organizational demand of the school
Changes in content								
A and D	English language in print and digital material	The material was translated to Spanish and the language style was fitted	Tailoring and refining	Preliminary adaptation design	Planned	Researchers, program manager, local stakeholders, intervention developers	To address cultural factors and to improve fit with the pupils	Local community members’ spoken language
A and D	Information on tobacco consumption, policies, and health care to smokers using UK data	Information on tobacco consumption, policies, and health care to smokers using Colombian data	Substituting and adding elements	Preliminary adaptation design	Planned	Researchers, program manager, local stakeholders	To preserve intervention outcomes and to improve fit with pupils’ engagement	Existing policies and historical context about tobacco
A	Graphical material (poster, information cards, and flyers) previously designed	All graphical material was redesigned to include the messages in Spanish and contextual images	Tailoring and refining	Preliminary adaptation design	Planned	Program manager, local stakeholders, intervention developers	To preserve intervention outcomes and to address cultural factors	Existing policies, historical context and cultural norms
A	Quiz activities at training and a follow-up session designed in the manual	New didactic material was designed (i.e., crossword puzzles)	Substituting elements	Adaptation refinement	Planned	Program manager, individual practitioners, and pupils	To increase pupils’ engagement and motivation	Pupils’ motivation and cultural norms regarding academic evaluation in a game context
A	Games and group dividing activities detailed in the manual	Local games and children’s rounds and songs were included	Substituting and adding elements	Preliminary adaptation design and test	Planned	Program manager, individual practitioners	To increase pupils’ engagement and motivation	Local practices and previous experience in similar interventions
D	Using Northern Irish examples of products, famous people, and social media	Using Colombian examples of products, famous people, and social media	Tailoring and refining	Preliminary adaptation design and adaptation refinement	Planned	Researchers, program manager, local stakeholders, individual practitioners, pupils	To improve fit with the pupils, to increase engagement and to preserve intervention outcomes	Pupils’ motivations, practices, knowledge, and cultural norms
D	Videos in English language with Northern Irish actors	Dubbed videos to Spanish using local accents	Tailoring and refining	Preliminary adaptation design	Planned	Researchers, program manager, local stakeholders, and intervention developers	To address cultural factors, to improve fit with pupils, and to reduce cost	Community members’ spoken language and availability of resources
D	Comparing tobacco with other psychoactive substances	Mentioning other substances was dropped	Removing elements	Preliminary adaptation design	Planned	Researchers, program manager, local stakeholders, intervention developers	To address cultural factors and policies	Existing policies, historical context and cultural norms about prevention of psychoactive substances use
Changes in training and evaluation								
A	Games and group dividing activities suggested in the manual	Focusing on using games and group dividing activities to manage the pupils’ energy	Adding elements	Adaptation refinement	Planned	Intervention developers, program manager	To improve the fit with pupils	Practitioners’ training and skills found out during the fidelity assessment
D	Teachers took a 120-min course before delivery	Individual practitioners took 7 hr of training before delivery	Substituting	Preliminary adaptation test	Reactive	Researchers, program manager	To preserve intervention outcomes and to improve the feasibility	Personnel modifications arise and compliance, organizational demand of the school
D	Reinforce skills planned on the manual	Focusing on strategies to reinforce pupils’ skills and the learning objectives of lessons	Adding elements	Adaptation refinement	Planned	Intervention developers, program manager	To preserve intervention outcomes	Practitioners’ training and skills found out during the fidelity assessment
Changes in implementation and scale-up								
A and D	Intervention developers offer the program to schools	Schools were selected supported by local government institutions	Adding elements	Preliminary adaptation design	Planned	Researchers, program manager, local stakeholders	To increase the feasibility of the program, to reach schools, and to engage schools	Organizational and competing demand of the schools. Historical context and social norms about education institutions

*A* ASSIST; *D* Dead Cool.

#### Changes of context

In ASSIST, the setting of the peer supporter training was an important contextual modification. The peer supporters who received training at the university’s campus reported that using this location resulted in a significant experience (Table 2). As one practitioner reported:

*They* [pupils] *are kids who do not have enough opportunities to access professional education. Most of them would finish school and maybe start a complementary technical cycle, or in the worst-case, the families force them to get a school certificate to start a job, but there is no motivation towards professional education. (…) How often does a group of children enter a private university and access different settings and activities as we planned here? This experience allows them* [children] *to imagine a potential scenario within their life project*. (Individual practitioner, interview, November 10, 2019).

Although the original design dictated that the peer supporter training should take place in a venue different from the school (e.g., hotels, conference rooms), using the university campus as a delivery location for the adapted programs was considered to be a relevant contextual modification because our location choice not only helped to make peer supporters feel “special” but it also helped them to imagine going to university in the future, which reflected an unplanned but positive outcome that inspired the pupils.

In Dead Cool, the most important contextual modification related to the personnel delivering the program. As the program was new in Bogotá, the teachers could not commit to delivering Dead Cool. We invited the counselor teachers to participate in the program, as they are accountable for prevention activities. However, they could not schedule the program within the classrooms because of work overload. As one practitioner commented:

*They* [teachers] *said they had many things to do and they did not want to assume additional responsibilities (…) I mean, they have multiple responsibilities in their everyday life and taking on the program, to do it well, implies that they need to have time to read it, understand it, prepare each lesson. Actually, I doubt they have the time availability to do it within the school*. (Individual practitioner, interview, July 11, 2019).

To ensure consistent outcomes and improve the program’s feasibility, a professional group with experience in school education (individual practitioners) was hired to deliver the program in the classrooms within the school schedule. This modification increased the time and content required for practitioner training (Table 2).

#### Changes of content

These modifications focused on adding elements or refining material to address cultural factors, to increase the feasibility and fidelity to preserve intervention outcomes. Most of the modifications were made during the adaptation refinement step, according to the suggestions of the intervention developers, participants and individual practitioners (Table 2).

In ASSIST, contributions from multiple roles helped to increase pupils’ engagement and to ensure that the peer supporters could sustain preventive conversations with their peers. Modifications to Dead Cool focused on adjusting the material to reflect existing local policies, laws, and social norms. In particular, we suppressed two elements to avoid iatrogenic outcomes [32]: (i) any image suggesting indirect promotion of tobacco consumption; and (ii) substance-specific content that was inappropriate for our target population. Intervention developers, pupils, and individual practitioners all contributed to increase the feasibility and improve the fit of the program. For example, we adapted the program using locally famous people, tobacco products, and social media. Supplementary Appendix 2 presents examples of content modifications.

#### Changes in training and evaluation

These modifications focused on adding elements to the training to preserve the intended outcomes and to improve the fit for the pupils. These adaptations were made according to the suggestions of the original developers during the fidelity assessment (Table 2).

#### Changes in implementation and scale-up

These modifications focused on increasing the acceptability of the interventions among the schools (Table 2). Furthermore, to facilitate potential future scale-up of the interventions, we involved local practitioners from the education and public health government offices to ensure adherence to existing guidelines, and provided training to teachers and stakeholders [33].

We encountered some challenges in engaging the participating schools and promoting the adoption of the interventions after the study. We focused on building human capacity in the local public health departments and the schools using the following strategies. First, to increase the trust of the school principals, we brought them together with the local education and public health secretaries, to introduce the implementation team and demonstrate their substantial qualifications and experience of working with adolescents. Second, to increase the interest of teachers and public health practitioners, we offered a professional development course on health behavior, certificated by the university. Third, we used high-quality materials (workbooks, toys, posters, cards, and manuals) to increase the interest of the teachers, pupils, and public health practitioners. Fourth, to increase the trust of parents whose children were acting as peer supporters (and were participating in training sessions outside of school premises), we invited them to information meetings and provided factsheets with phone numbers. Fifth, to build capacity for scale-up, practitioners from the local public health and education offices participated in the training and the professional development course.

### Findings of the cultural adaptation trial (step 5)

ASSIST activities were implemented with a group of peer supporters in each school (*N* = 3 groups). The nine sessions of Dead Cool were implemented in all classes of each school (*N* = 10 groups). Table 1 presents the sociodemographic characteristics of the participants taking part in step 5.

#### Fidelity of the implementation of the programs

The fidelity assessment provided by the original program developers had a positive score for both programs. In addition, the self-reported fidelity monitoring showed that 86% of the ASSIST activities and 88% of the Dead Cool activities were delivered as per the protocol guides. The qualitative analysis showed that problems with the delivery were related to the availability of time and technical resources in situ. These were managed by individual practitioners who were able to cover the missing themes.

#### Outcomes

The trial indicated that pupils in ASSIST schools (OR = 2.00 [95% CI: 1.21–3.30]; *p* = .007) and pupils in Dead Cool schools (OR = 1.77 [95% CI: 1.13–2.77]; *p* = .013) were more likely to increase their knowledge of smoking after the interventions compared with the baseline measurement. Additionally, among the pupils who had never smoked at baseline, 92.3% of ASSIST pupils and 86.9% of Dead Cool pupils retained the status of “never smoker” after the intervention.

#### Engagement of the pupils

The trial indicated that the participants engaged well with the programs. On average, the peer supporters attended 87% of the ASSIST activities, whilst the Dead Cool pupils attended 90% of the activities. Furthermore, after the interventions the proportion of students reporting that the school had given them enough information on smoking increased by 12.5 percentage points in ASSIST schools (38.8% at baseline and 51.3% postintervention; *p* < .001) and 17.3 percentage points in Dead Cool schools (48% at baseline and 65.3% postintervention; *p* < .001).

The qualitative analysis also showed the pupils engaged well with both programs. First, the ASSIST peer supporters highlighted the skills learned and their roles with their peers. As one peer supporter said:

*This experience was very enjoyable. Before, I didn’t know what a cigarette contains. Now, I know what it is made of and what its health effects are. (…) I felt good because we did help some smoker classmates, and if they didn’t smoke, told them not to do it. I told them all the things that a cigarette contains*. (Peer supporter, focus group, October 24, 2019).

This comment illustrates that the program helped the peer supporters to develop the skills they needed to communicate smoking prevention messages to their peers who had intentions to smoke. The cultural adaptation process of ASSIST ensured the content reflected the pupils’ needs and the schools’ context.

Second, the Dead Cool pupils emphasized the skills learned during the program. One pupil said:

*In Bacanísimo, teachers teach us the risks of tobacco. It was a change for me because before the program I had the thought that as soon as I turn eighteen I was going to smoke my first cigarette. But, after finding out that the cigarette has consequences, well I don’t want to do it*. (Pupil, focus group, October 1, 2019).

As this comment illustrates, the program helped to achieve a change in the pupils’ attitudes toward tobacco, while they developed the skills to reject it. Thus, the cultural adaptation process for Dead Cool ensured that the content was tailored to pupils’ needs and the schools’ context.

## DISCUSSION

This is one of the first studies to report the systematic cultural adaptation of evidence-based smoking prevention programs in LA. We adapted two evidence-based programs, originally developed in the UK, to prevent tobacco consumption in schools in Bogotá using the FRAME model. This framework reflects the complexities and dynamics of different settings for implementation, and permits the replication of the cultural adaptation process in different contexts [24]. Our study incorporates the local context in the content and the implementation strategy, while monitoring the fidelity of the original programs. According to the FRAME model, the modifications were fidelity consistent because they were planned to preserve the core elements of the interventions that are needed to maintain the intended outcomes and increase their feasibility in the local context [24]. These core elements were identified early on by local stakeholders and the original program developers. This approach was completed in a way that could be replicated in other countries of LA and the global south.

Previously, some substance use prevention interventions have been adapted for the Latino population living in the USA [34]. However, researchers have argued that prevention programs are more successful when they account not only for the culture and ethnicity of the target population, but also for contextual aspects, to ensure engagement and sustainability [35]. In addition, the literature suggests that it is important to take account of factors in the local public health structure, implementation practices, and culture, to facilitate the scaling-up of interventions in new settings [33]. Our findings highlight the importance of incorporating different roles in the cultural adaptation process and collaborative research. Involving the original program developers maintained the fidelity of the implementation of the programs. Meanwhile, involving stakeholders and local government officers supported the engagement of the schools and allowed for adjustment of the content to be more consistent with local policies and practices, complying with the best emergent practice [26, 36]. Involving pupils, teachers, and individual practitioners is recommended to ensure engagement and to identify issues and solutions around feasibility. Involving practitioners from the local public health and education structure means there is potential for both adapted programs to be adopted on a larger scale in Bogotá in the future [33, 36].

Our experience of conducting the cultural adaptation allows us to reflect on the successful aspects of intervention fidelity, outcomes, and engagement of the culturally adapted programs. This provides an opportunity for future researchers to consider further empirical evaluations of the effectiveness and applicability of these evidence-based interventions in other local contexts [37, 38]. Previously, ASSIST and Dead Cool have been shown to reduce the proportion of adolescents who start smoking compared with a control group [12, 13]. The adapted programs have common aims but derive from two different theoretical approaches. ASSIST is based on a social influence approach and Dead Cool is based on personal skills learning and the Theory of Planned Behaviour. Studying these two approaches might enhance behavior change among adolescents because they target different influences operating at intrapersonal and interpersonal levels [18].

Therefore, our findings provide an evidence-based strategy to promote awareness of tobacco control within school environments in Colombia, contributing to the global effort toward tobacco control. At the international level, delivering culturally adapted versions of intervention programs broadens the access to effective and comprehensive education on health risk, in accordance to the WHO-FCTC recommendations [39]. At the national level, school-based smoking prevention aligns with existing intersectoral tobacco control policies that prioritize healthy school environments [21]. At the local level, the outcomes of the intervention programs can contribute to the prevention of chronic diseases and the promotion of a healthy lifestyle among adolescents and their families as part of the government’s development plan.

Our study has some limitations. We did not set out to evaluate the interventions’ effectiveness and long-term adoption (since this has been shown in previous Randomized Controlled Trials in the UK), but rather focused on their implementation. This is because the main objective of the *MECHANISMS* study is to uncover the potential behavioral mechanisms of action of the programs, rather than to provide a “head-to-head” comparison of their effectiveness. However, there is little consensus on when an adapted intervention needs a full reevaluation of its effectiveness. Only one of a growing number of adaptation frameworks has attempted to address this question and, not surprisingly, concluded that the degree of reevaluation required will depend on the extent of changes made to core intervention components and on the similarity or distinctiveness of the new context [40]. Other frameworks have emerged from the epidemiological and public health traditions [37, 41] that might be usefully drawn upon to assess the transferability of evidence in new settings. Whilst we believe that this study will help future researchers assess how to adapt ASSIST and Dead Cool to the cultural context of Colombia, our participant population was drawn from official school rolls, which represent 59% of the enrolled population in the city and 77% of the country [17]. Some elements of the programs might need to be adapted to slightly different contexts of private schools and other cities before scaling-up to national level. Future research could assess the potential for long-term adoption of the interventions in complex settings.

In our discussions as a research team, we deliberated upon the often little-acknowledged difficulty of defining the “functional” fidelity of “core” components when it usually has to be taken on faith that a program theory (and the associated logic model) can be activated in a new context. A realist or complex systems thinker might doubt that intervention components can be bounded at all, or at least bounded in the same way for all [42]. Indeed, whilst we acknowledge the progress made recently on the development of typologies for logic models, including how they consider context [43], one might argue that context and program theory are reciprocally generative, like the compounds in a chemical reaction that might or might not be catalyzed in different conditions or at different temperatures. The notion of a single coherent intervention theory might then be “fool’s gold.” The added benefit, and a transferable lesson, from the process of adapting these two interventions, was that we began to raise such questions. Even if they have no simple answer, they speak to the need for multinational research teams such as ours to showcase reverse innovation where learning can be transferred from low to high resource settings [44].

## CONCLUSION

This study carried out the cultural adaptation of two school-based smoking prevention programs, and contributes to the translation of evidence-based interventions for health-related behaviors in a city from a LMIC. Researchers and decision makers could use these results and methodology to replicate the interventions in other settings of LA, and the global south. The results assert the need to broaden the access to context-adjusted education on health risks in the context of the WHO-FCTC.

## Supplementary Material

ibab019_suppl_Supplementary_Appendix_1

ibab019_suppl_Supplementary_Appendix_2
