# Supplementary material for: Cultural adaptation of two school-based smoking prevention programs in Bogotá, Colombia
Source: Transl Behav Med. 2021 Apr 26;11(8):1567–78. doi: 10.1093/tbm/ibab019 (PMC8499713; doi:10.1093/tbm/ibab019)
Supplement: ibab019_suppl_Supplementary_Appendix_1 [file ibab019_suppl_supplementary_appendix_1.docx]

**Supplementary Appendices**

**Cultural adaptation of two school-based smoking prevention programs in Bogotá, Colombia**

| **Supplementary Appendix 1.** Activities for culturally adapting ASSIST and Dead Cool. | | |  |  |
| --- | --- | --- | --- | --- |
|  |  |  |  |  |
| **Step name** |  | **Activities involved** |  | **Participants involved** |
|  |  |  |  |  |
| 1. Information Gathering |  | - Build a local stakeholder committee. |  | Researchers, Program manager, Intervention developers, Local expert committee. |
|  |  | - Identify relevant contextual factors, and social and cultural values. |  |  |
|  |  | - Identify interventions related, risk behaviours and environmental factors in the context. |  |  |
|  |  | - Identify the organizational capacity and target population. |  |  |
|  |  | - Determine whether the intervention goals and outcomes are relevant to the target populations. |  |  |
|  |  | - Obtain and review the original intervention materials. |  |  |
|  |  | - Examine the theory base behind the interventions, mechanisms of change, moderators, and outcomes. |  |  |
|  |  | - Training on both interventions to identify the core components and best-practices characteristics. |  |  |
|  |  | - Determine the possible ways to adapt and fit to the needs of the local context. |  |  |
|  |  | - Define the objectives of the adaptation process. |  |  |
|  |  |  |  |  |
| 2. Preliminary adaptation design |  | - Develop an adaptation plan. |  | Researchers, Program manager, Intervention developers, Local stakeholders committee. |
|  |  | - Identify potential mismatches and barriers to implementation and participation. |  |  |
|  |  | - Develop a strategy for achieving fidelity of implementation. |  |  |
|  |  | - Translation of all the intervention materials (English to Spanish). |  |  |
|  |  | - Dubbing voice of the videos (English to Spanish). |  |  |
|  |  | - Adapt to the local language to be understandable and culturally appropriate. |  |  |
|  |  | - Back translation using interpretative sense-checking (Spanish to English). |  |  |
|  |  | - Gathering local pedagogical and informational materials. |  |  |
|  |  | - Assess stakeholders input and potential collaborations.  - Invite the local education and public health practitioners to participate. |  |  |
|  |  | - Consider the intervention training and organization staff. |  |  |
|  |  | - Develop a “mock-up” version of the adapted materials. |  |  |
|  |  | - Prepare design documents and user-friendly manuals of the interventions. |  |  |
|  |  | - Discuss with the intervention developers and the local expert committee about the discrepancies. |  |  |
|  |  | - Adapt the relevant intervention components through collaborative efforts. |  |  |
|  |  | - Label the adapted version of ASSIST as *Entre Parceros*, and Dead Cool as *Bacanísimo*. |  |  |
|  |  |  |  |  |
| 3. Preliminary adaptation test |  | - Develop an implementation plan. |  | Researchers, students, intervention developers, individual practitioners, program manager, community members. |
|  |  | - Contact local stakeholders and decision-makers.  - Establish a collaboration agreement with local education and public health practitioners. |  |  |
|  |  | - Recruit the intervention team and train individual practitioners.  - Train individual practitioners on the study. |  |  |
|  |  | - Collect socio-demographic information using a survey. |  |  |
|  |  | - Deliver *Entre Parceros* and *Bacanisimo.* |  |  |
|  |  | - Register the implementation of each activity on the field diaries. |  |  |
|  |  | - Monitor the fidelity of the intervention delivery by the intervention developers. |  |  |
|  |  | - Conduct interviews and focus groups to individual practitioners and students. |  |  |
|  |  |  |  |  |
| 4. Adaptation refinement |  | - Refine adaptations based on results generated in previous steps. |  | Researchers, individual practitioners, program manager |
|  |  | - Correct and optimize all aspects in which difficulties were encountered. |  |  |
|  |  | - Establish ongoing support, feedback and refinement. |  |  |
|  |  |  |  |  |
| 5. Cultural adaptation trial |  | - Implement the adapted interventions within six schools with random assignation of each intervention. |  | Researchers, students, individual practitioners, program manager, community members. |
|  |  | - Recruit the intervention team and train individual practitioners.  - Train individual practitioners, and local education and public health practitioners on the study. |  |  |
|  |  | - Collect socio-demographic information using a survey. |  |  |
|  |  | - Delivery of *Entre Parceros* and *Bacanisimo.* |  |  |
|  |  | - Conduct interviews and focus groups to individual practitioners and students. |  |  |
|  |  | - Assess the acceptance of and participants’ engagement in the adapted intervention. |  |  |
|  |  | - Establish ongoing support, feedback and refinement.  - Offer a professional development course to teachers, and local education and public health practitioners. |  |  |
|  |  | - Revise the intervention by recommending to adopt effective or drop ineffective adaptations. |  |  |
