# Supplementary material for: Cultural adaptation of two school-based smoking prevention programs in Bogotá, Colombia
Source: Transl Behav Med. 2021 Apr 26;11(8):1567–78. doi: 10.1093/tbm/ibab019 (PMC8499713; doi:10.1093/tbm/ibab019)
Supplement: ibab019_suppl_Supplementary_Appendix_2 [file ibab019_suppl_supplementary_appendix_2.docx]

**Supplementary Appendices**

**Cultural adaptation of two school-based smoking prevention programs in Bogotá, Colombia**

| **Supplementary Appendix 2.** Examples of key content adaptations of ASSIST and Dead Cool. |  |  |
| --- | --- | --- |

1. Tailoring and refining graphic material in the ASSIST adaptation

| **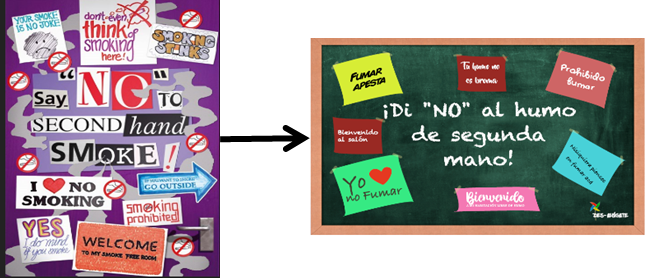** | |
| --- | --- |
| Original version | Adapted version |

1. Substituting elements regarding local policies in the ASSIST adaptation

| 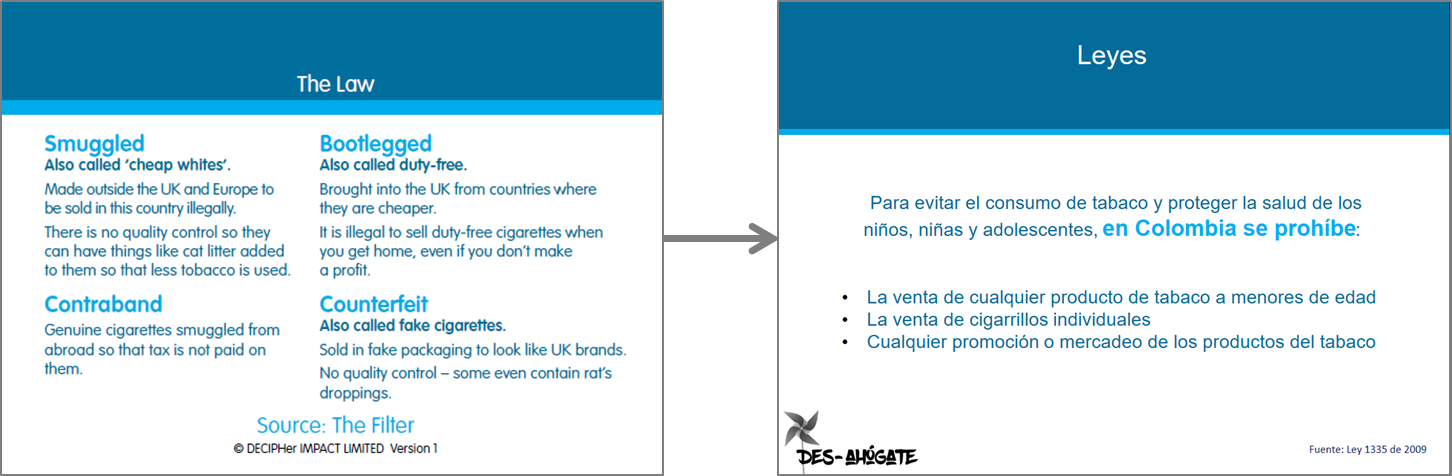 | |
| --- | --- |
| Original version | Adapted version |

1. Removing elements regarding other substances in Dead Cool

| 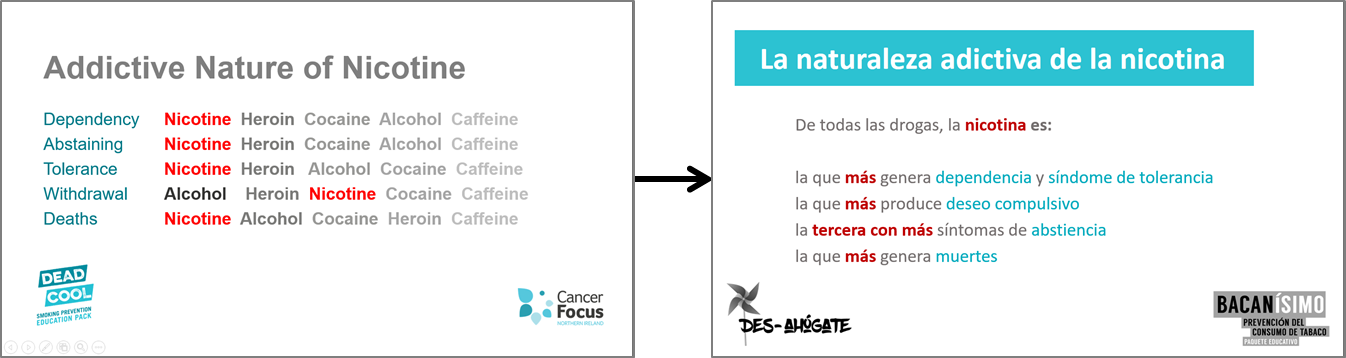 | |
| --- | --- |
| Original version | Adapted version |

1. Substituting elements according to pupils’ suggestions in Dead Cool

| 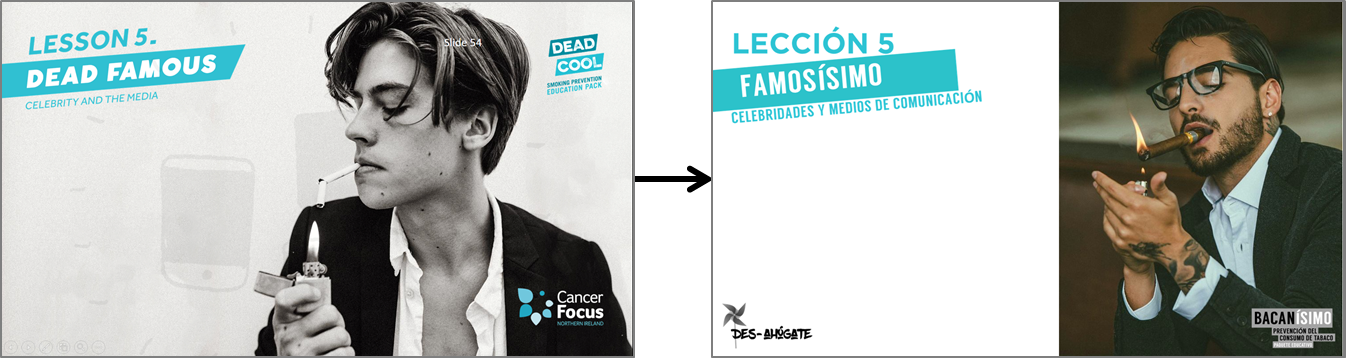 | |
| --- | --- |
| Original version | Adapted version |

**c.** Including new material according to the pupils’ interest

| 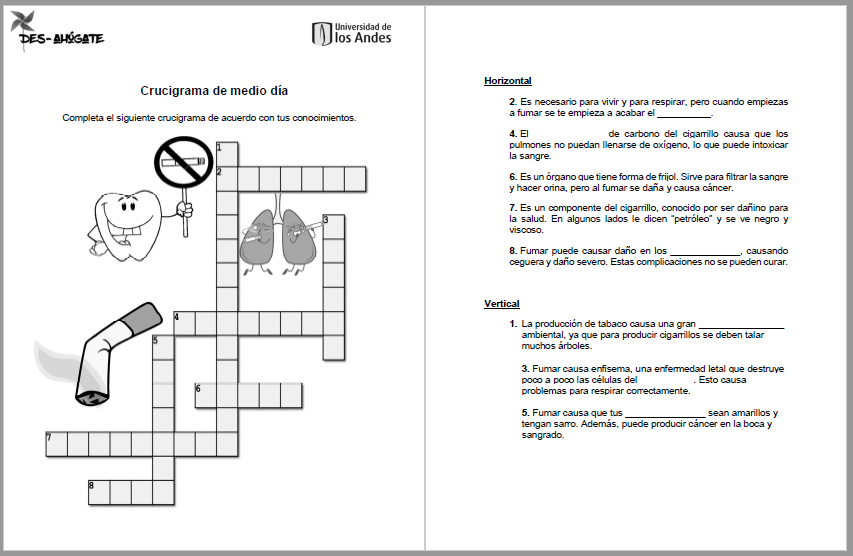 |
| --- |
